# Effect of Tree Density on Yield and Fruit Quality of the Grafted Hazelnut Cultivar ‘Tonda Francescana^®^’

**DOI:** 10.3390/foods13203307

**Published:** 2024-10-18

**Authors:** Silvia Portarena, Simona Proietti, Stefano Moscatello, Claudia Zadra, Nicola Cinosi, Chiara Traini, Daniela Farinelli

**Affiliations:** 1 Institute of Research on Terrestrial Ecosystems (IRET), National Research Council (CNR), Via G. Marconi 2, 05010 Porano, Italy; simona.proietti@cnr.it (S.P.); stefano.moscatello@cnr.it (S.M.); 2 National Biodiversity Future Center (NBFC), Piazza Marina 61, 90133 Palermo, Italy; 3 Department of Pharmaceutical Sciences, University of Perugia, Via del Giochetto-Ed. B, 06121 Perugia, Italy; claudia.zadra@unipg.it; 4 Department of Agricultural, Food, and Environmental Sciences (DSA3), University of Perugia, Via Borgo XX Giugno 74, 06121 Perugia, Italy; nicola.cinosi@unipg.it (N.C.); chiara.traini@dottorandi.unipg.it (C.T.); daniela.farinelli@unipg.it (D.F.)

**Keywords:** corylus avellana, planting density, light penetration, yield, fruit quality, monounsaturated fatty acids (MUFA), α-tocopherol, nonstructural carbohydrates, protein, fat content

## Abstract

Optimizing planting density is crucial for balancing resource competition, light penetration, and tree productivity in orchard systems. This study investigateed the impact of planting density on the yield and fruit quality of the hazelnut cultivar ‘Tonda Francescana^®^’ grafted onto *Corylus colurna* L. rootstocks. The research aimed to assess how different planting densities influenced light penetration, canopy volume, yield, and the nutritional profile of hazelnuts during their sixth growing season. Three planting densities were tested: 625, 1250, and 2500 trees per hectare (low, medium, and high density, respectively). The results show that medium-density planting provided the best balance between light availability, canopy development, and yield efficiency. The synthesis of monounsaturated fatty acids (MUFA) and α-tocopherol (vitamin E) was more prominent in the medium-density system (80.2% and 10.3%, respectively), suggesting a favorable metabolic response to moderate competition for resources. In contrast, high-density planting yielded the most per hectare (2898 kg/ha) but exhibited lower individual tree productivity (1.16 kg). Low-density planting had the highest light penetration (53%) but lower overall yield (822 kg/ha) and quality, with greater starch accumulation in the fruit. In general, medium-density planting optimized both yield and kernel quality, with potential implications for orchard management and breeding strategies to enhance hazelnut production and nutritional value.

## 1. Introduction

The European hazelnut (*Corylus avellana* L.) is a species of significant interest for its nutritional value, with global cultivation covering more than 660,000 hectares worldwide. Major producers include Turkey, Italy, the USA, Georgia, Azerbaijan, and Spain, but cultivation has expanded to new regions in the Southern Hemisphere, such as Chile, South Africa, and Australia [1]. Hazelnuts are recognized for their high content of healthy fats, proteins, dietary fiber, and essential nutrients. In particular, they are rich in unsaturated fatty acids like linoleic, linolenic, and oleic acids, which contribute to cholesterol reduction and improved cardiovascular health [2,3]. Additionally, the α-tocopherol (vitamin E) content is linked to a reduced risk of chronic conditions, such as heart disease, type 2 diabetes, hypertension, and cancer [4,5].

The yield and quality of hazelnuts, like other horticultural crops, are influenced by a combination of environmental factors, genotype factors, and management practices [6,7,8,9]. Among these factors, plant density has emerged as a crucial element for optimizing yield and kernel quality [10,11]. Higher planting densities can lead to intensified competition among trees for essential resources such as light, water, and nutrients, which may negatively affect nut size and overall quality [12]. However, when properly managed, increased planting densities can yield higher production per hectare due to the greater number of trees, potentially leading to increased total nut production, provided that the trees do not experience excessive stress [13,14].

Key quality parameters, including kernel weight, fat content, and fatty acid composition are critical for determining the commercial value of hazelnuts. Heightened competition at higher planting densities often results in reduced kernel sizes and lower fat content due to resource limitations, adversely impacting the flavor profile and texture of the nuts [15]. Ensuring an optimal planting density allows for better light penetration, nutrient uptake, and air circulation, which are essential for developing high-quality kernels [14,15,16]. Achieving a balance between tree spacing and resource availability is vital for maintaining desirable kernel characteristics, such as fat content and fatty acid composition, without compromising yield.

While some research has explored the effects of tree density on hazelnut productivity, most studies, such as those by [10,16], have focused on self-rooted hazelnuts. To date, no studies have investigated hazelnuts grafted onto low or non-suckering rootstocks like *Corylus colurna* L., which could significantly reduce the need for desuckering—an often-challenging task, particularly in high-density orchards [17,18,19]. The current study aims to address this gap by examining the impact of various planting densities on the yield and quality of grafted ‘Tonda Francescana^®^’ hazelnuts cultivar using *Corylus colurna* L. rootstocks. This approach could mitigate the challenges associated with sucker control, offering valuable insights into orchard management under high-density conditions.

By investigating the influence of planting density on light availability, nut quality, and yield, this research seeks to contribute to improved crop management practices and breeding strategies [20,21,22]. By focusing on these interactions, the research will also employ chemometric analysis to characterize quality traits, allowing for the identification of superior fruit characteristics. This approach will deepen our understanding of how planting density affects hazelnut quality and its potential relevance to overall health.

## 2. Materials and Methods

### 2.1. Orchard Characteristics and Weather Data

This study took place during the 2022 growing season in an experimental orchard managed by the Department of Agricultural, Food, and Environmental Sciences at the University of Perugia, in central Italy (42°58′22.82″ N, 12°24′13.02″ E). The orchard was established in 2016. It featured three rows with different planting densities: 625 trees per hectare (low density), spaced 4 m between and within rows; 1250 trees per hectare (medium density), with 4 m between rows and 2 m within rows; and 2500 trees per hectare (high density), with 4 m between rows and 1 m within rows. The soil was loamy, with balanced proportions of sand (38.4%), silt (38.4%), and clay (23.2%), providing a good balance of drainage and moisture retention. The high pH (8.2) reflected alkaline conditions, influenced by the high total limestone content (18%). Both the phosphorus (43 ppm) and nitrogen (0.73%) levels in the soil are relatively low, so these nutrients were applied in spring to meet the plants’ needs during the growing season. Organic matter (1.3%) as also low, so organic fertilizers were added in autumn to help improve soil fertility. The soil was managed through mechanical tilling under the rows, while grass was maintained between the rows and periodically mowed to keep the orchard clean and minimize competition for water and nutrients.

The trees, of the Tonda Francescana^®^ cultivar grafted onto a no-suckering rootstock from *C. Colurna*, were pruned to a single trunk with three to four main branches. Sub-irrigation was provided from mid-May to the end of August, depending on seasonal weather conditions. Meteorological data were collected using a Spectrum Watch-Dog 2000 Series (Spectrum Technologies Inc., Aurora, IL, USA) Weather Station located near the orchard. The temperature was recorded at a time resolution of 15 min.

### 2.2. Canopy Volume, Yield and Light Penetration in the Canopy

Tree height, trunk height, and canopy dimensions (height, width, and thickness) were measured by a trained operator using a standard measuring tape while standing in front of each tree. Canopy height was determined by subtracting the trunk height from the total tree height. Canopy width and thickness were calculated as the average distances between opposite sides of the canopy. Given that the rows of hazelnut trees were oriented north–south, the width was measured as the average distance between the north and south sides of the canopy, while the thickness was measured between the east and west sides. These measurements of width and thickness were used to calculate the average canopy radius, and together with the canopy height, the canopy volume was estimated by approximating it as a cylinder [23].

At the end of August, the yield was determined by collecting all the nuts from each plant. The hazelnuts were gathered from the ground using a FACMA MEK 1800 ( FACMA S.R.L., Viterbo, Italy) mechanical raking machine. The nuts were then sorted by tree density and row to ensure accurate yield measurements. Yield efficiency was calculated by dividing nut yield (kg) by the canopy volume (m^3^) and expressed as kg·m^−3^.

In July, the radiation intercepted by the tree was measured manually using a luxmeter (AccuPAR, Decagon Devices Inc., Pullman, WA, USA). This device features a movable bar that can slide to various positions along the horizontal axis, ranging from 0 cm (ground level) to 50 cm or 100 cm, and can also be adjusted vertically with a screw mechanism, extending from 0 cm (ground) to 150 cm. This setup allowed for the measurement of light intensity at different heights within the plant, which could then be compared to light levels in full sunlight, thereby indicating the light intercepted by the tree canopy. To estimate the overall light interception by the entire canopy, the mean value of all recorded data taken at various distances from the trunk and at different heights of the tree was calculated [23].

### 2.3. Fruit Protein, Fat Content and Non-Structural Carbohydrates (NCS) Analysis

At the end of August 2022, fruit samples were collected. For each planting density, six plants were randomly selected, resulting in a total of 18 plants involved in the experiment. From each sample, 20 kernels were freeze-dried to a constant weight. Dried samples were ground into a fine powder using a mortar and pestle, with liquid nitrogen applied throughout the grinding process. The kernel nitrogen (N) content was analyzed using an elemental analyzer (Model NA 1500, Carlo Erba, Milan, Italy) and expressed as a percentage (%) of the dry matter. The total protein content was obtained from the total N concentration by applying a conversion factor of 6.25 [24].

Total fat content was determined by extracting 1 g of the dried kernel with hexane using a Buchi extraction system B-811 (Büchi Labortechnik AG, CH-9230 Flawil 1, Flawil, Switzerland) and applying the parameters of the extraction program described in Buchi Application Note 003-411.

Non-structural carbohydrates (NSC) in kernels were analyzed by extracting 10 mg of lyophilized powder in 80% ethanol at 80 °C for 45 min with constant shaking. The extract was then centrifuged (Centrifuge 5415R, Eppendorf AG, Hamburg, Germany) at 16,000× *g* for 5 min. Soluble sugars (glucose, fructose, and sucrose) were collected from the supernatant, while starch remained in the pellet. A spectrophotometric enzymatic assay was used for the quantification of both soluble sugars and starch, following the method in [25]. The starch-containing pellet was washed four times with 50 mM sodium acetate buffer (pH 4.5), resuspended, and autoclaved at 120 °C for 45 min in 1 mL of the same buffer. The sample was then incubated at 50 °C for 1 h with amyloglucosidase (70 U) and α-amylase (4 U) to hydrolyze the starch into glucose, which was quantified using a spectrophotometric enzymatic assay as previously described.

### 2.4. Tocoferol Determination

Hazelnut oil was extracted from nut samples, and tocopherols were measured using a modified version of the method from [26]. The extraction process used 200 mg of lyophilized hazelnut powder mixed with 2 mL of hexane. The samples were vigorously vortexed for 1 min and then placed in an ultrasonic bath at 40 °C for 20 min. The hazelnut extract was centrifuged at 10,000× *g* for 10 min, and the pure hazelnut oil was obtained after the evaporation of the organic layer under vacuum at 45 °C for 45 min. For tocopherol analysis, 100 mg of hazelnut oil was weighed and extracted with 400 μL of isopropanol. The mixture was vortexed for 30 s and centrifuged at 10,000× *g* for 10 min, then filtered using 0.2 μm nylon syringe filters (GE-Whatman, Maidstone, UK) and injected into the HPLC system.

Tocoferol was quantified as a sum of α-Tocoferol + γ-Tocoferol using an HPLC U3000 system (DionexTM Ultimate 3000 HPLC; Thermo Fisher Scientific, Waltham, MA, United States), equipped with a C18(2) LUNA analytical column (5 μm, 250 mm × 4.6 mm) and a related guard column (all by Phenomenex, Bologna, Italy) maintained at 30 °C. The mobile phase consisted of (A) methanol (MeOH) and (B) acetonitrile (ACN), following a gradient of 50% (A) and 50% (B) for the first seven minutes (*t* = 0–7), then increasing to 95% (A) and 5% (B) over the next five min (*t* = 7–12), and holding at 95% (A) for an additional three min (*t* = 12–15). The initial condition of 50% (A) and 50% (B) was restored for 15 min (*t* = 15–30). The flow rate was set at 1.0 mL/min, and the autosampler was kept at 4 °C. Tocopherol concentrations were measured at a UV wavelength of 295 nm using standard curves. Both the eluents and standard solutions of α-tocopherol and γ-tocopherol were prepared with HPLC-grade reagents from Merck KGaA (Darmstadt, Germany). The chromatography system, U3000-HPLC, was controlled, and data were acquired and processed using Chromeleon Data System 7.1 software (Dionex™ ICS-5000; Thermo Fisher Scientific, Waltham, MA, USA).

### 2.5. Fatty Acid (FA) Determination

The fatty acid (FA) composition was determined by converting the FAs into their corresponding methyl esters. The transmethylation process, gas chromatography with a flame ionization detector (GC-FID) (Trace 2000; Thermo-Fisher), and the specific analytical conditions were applied as described by [27]. The relative amounts of individual FAs were calculated based on their chromatographic peak areas, and the percentage of each FA was determined in relation to the total peak areas of both saturated and unsaturated FAs.

### 2.6. Statistical Analysis

Statistical analysis was performed by means of one-way ANOVA, with the plant density (low, medium, and high) as a factor. Differences between averages were tested by Fisher’s post hoc test, with a significance level of α = 0.05.

Principal component analysis (PCA) was performed using the fruit quality parameters as input variables to explore the variability among samples and to detect the most discriminating variables.

PCA summarizes the information contained in the data matrix in fewer independent PCs, obtained as linear combinations of the original variables, lying in the direction of maximum variance [28,29].

The data were statistically evaluated using the STATISTICA 10 software (StatSoft Inc.) and R 4.4.1 (R Core Team, 2024).

## 3. Results and Discussion

### 3.1. Experimental Orchard and Weather Data

**Figure 1:**
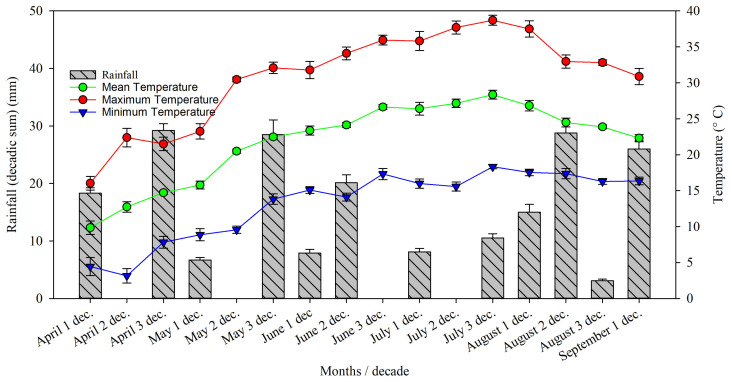
Amount of rainfall and mean, maximum, and minimum temperature recorded per decade from April to September 2022.

The climate data were reported from April to September, divided into ten-day intervals (Figure 1), as this period corresponds to the phase of canopy growth, development, and maturation/ripening of hazelnuts [30]. During these months, environmental conditions play a crucial role in the plant’s vegetative cycle, determining the quality and quantity of the harvest.

Throughout the recorded period, temperatures showed a clear upward trend as the season progressed. Maximum temperatures rose from around 16 °C in early April to peak at 38.7 °C in July. July and August were marked by extreme heat, with maximum temperatures consistently exceeding 35 °C, reaching a high of nearly 39 °C. Minimum temperatures also increased significantly, with night temperatures staying above 15 °C from June onward. This indicated a hot summer, particularly in July and August, which could lead to heat stress for hazelnut trees, impacting nut quality and kernel development. Precipitation fluctuated, with sporadic rainfall throughout the months. April had moderate rainfall, but May through July saw increasingly dry conditions, with several periods of zero rainfall, particularly in July. Although August experienced slightly more rainfall, particularly in the second decade, it remained relatively dry overall. In this context, increasing plant density might serve as a potential strategy to mitigate the effects of high temperatures and excessive sunlight on hazelnut trees, as denser canopies can provide shade and reduce heat stress [31].

### 3.2. Canopy Volume, Yield and Light Penetration in the Canopy

The analysis of canopy volume, light penetration, and yield provided valuable insights into how tree spacing influenced canopy development, light interception, and yield (Table 1).

In the high-density planting system with an averaged canopy volume of 3.5 m^3^, the percentage of light penetration into the canopy was significantly lower, at 19.2%. This reduction in light availability likely resulted from greater tree crowding, which limited sunlight reaching the inner canopy layers. Yield per tree in this system was also the lowest at 1.16 kg, resulting in a yield efficiency of 0.34 kg/m^3^. Despite the lower yield per tree, the overall yield per hectare was the highest at 2898 kg/ha due to the higher tree population compensating for individual tree productivity. In the medium-density planting system, the canopy volume was the largest at 4.4 m^3^, with 46.9% light penetration. This tree density, at the sixth year in the field, exhibited the highest yield per tree, with 1.67 kg/tree, and the highest yield efficiency at 0.39 kg/m^3^, suggesting a more optimal balance between canopy development and light interception. The total yield per hectare in the medium-density system was 2089 kg/ha. Although this was lower than the yield in the high-density system, it demonstrated a more efficient use of available resources for productivity. In comparison, despite having twice the number of trees per hectare, the high-density planting system produced only 38.7% more yield, underscoring the limitations in light availability and resource conversion efficiency at higher densities [15]. Finally, in the low-density planting system, the canopy volume was 3.7 m^3^, and light penetration was the highest, at 53%. While this increased light availability may have enhanced certain physiological processes, the yield per tree was moderate, at 1.32 kg, and the yield efficiency was similar to that in the high-density system, at 0.36 kg/m^3^. The overall yield per hectare was significantly lower at 822 kg/ha, primarily due to the reduced number of trees per unit area, despite the relatively higher light availability per tree.

### 3.3. Fat, Protein and NSC Contents

Statistical analysis revealed that both protein and fat contents were still not significantly influenced by the density of planting (Table 2). This indicated that variations in planting density, during the sixth growing season, did not have a meaningful impact on the protein or fat content of the hazelnuts, suggesting that other factors, such as tree genetics or overall environmental conditions, may have a more significant impact on these nutritional components than canopy structure or light availability.

This situation might occur because the trees still have sufficient space, both in width and height, to allow for adequate canopy development and light penetration [20,21,32]. Additionally, in high-density planting systems, fruiting tends to occur in the middle to upper parts of the canopy [16], which may explain the lack of significant qualitative differences and the modest increase in production compared to the medium-density planting system.

The consistent protein content across the three tree densities could be attributed to the fact that all hazelnuts, regardless of density, are positioned near leaves that still receive adequate light [22].

The analysis of NSC accumulation across the three planting densities provided insights into the modulation of metabolic responses under varying growth conditions. In specific, both glucose and starch were significantly affected by the density of planting, as reported in Table 2.

The glucose and sucrose concentrations were favored at medium density, which appears to strike a balance between excessive competition (high density) and under-utilization of resources (low density). The moderate plant competition at this density likely promoted optimal light penetration and resource allocation, which enhanced photosynthetic activity and sugar production.

The lower levels observed at a high planting density could be due to the increased competition for light, nutrients, and water, leading to limited photosynthesis and sugar synthesis. Conversely, at a low planting density, the decrease in glucose and sucrose may reflect a state where photosynthetic activity is not fully maximized due to the reduced shading effect, which could cause stress from excess light or heat exposure [8].

No statistically significant differences were found in the accumulation of raffinose, stachyose, and verbascose among the three planting densities. Their relatively stable concentrations across planting densities may suggest that these compounds are primarily regulated by other abiotic conditions (e.g., drought stress) rather than plant competition or light availability related to planting density [33].

Interestingly, starch content was significantly higher at low density, indicating that plants at this density accumulated more storage carbohydrates. This may reflect reduced metabolic demand for growth or stress-induced starch accumulation due to less competition for water and nutrients. The high starch levels at low density, along with stable raffinose, stachyose concentrations and verbascose, suggested that excess carbohydrates were not fully metabolized for immediate energy needs but were rather stored for future use [19]. On the other hand, high and medium planting densities showed lower starch accumulation, likely due to the active utilization of glucose and sucrose for growth and survival under more competitive conditions.

### 3.4. FA Composition

The FA composition of hazelnut oil, including saturated fatty acids (SFAs), monounsaturated fatty acids (MFAs), and polyunsaturated fatty acids (PUFAs), significantly varied across planting densities (Table 3).

The percentage of SFAs was significantly higher in the medium- and low-density planting system and lower in the high-density planting system. This variation in SFAs content can be attributed to environmental factors, such as light and temperature which influence the activity of enzymes involved in fatty acid synthesis. In the high-density planting system, individual plants receive less sunlight (Table 1), leading to reduced temperatures within the plant. Lower temperatures are associated with decreased synthesis of saturated fatty acids, as observed in previous studies [34,35].

In contrast, PUFAs showed the opposite trend to MUFAs, with the highest percentage of PUFAs observed in the high-density planting system. This suggested that crowding and reduced light penetration promote the synthesis of PUFAs. Under shaded conditions, plants often increase PUFA production as part of their stress response. PUFAs, being more reactive, play a critical role in maintaining membrane fluidity and contribute to plant defense mechanisms during light stress [34,35].

In conditions of optimal sunlight, as seen in the medium-density planting system, the plant’s energy production is maximized, favoring the synthesis of MUFAs and SFAs. Adequate light enhances photosynthesis and carbohydrate production, which can be efficiently directed toward oil synthesis. This results in a balanced fatty acid profile, where MUFA and SFA synthesis are more prominent due to higher energy availability.

From the perspective of shelf life, the type of fatty acid plays a crucial role. SFAs and MUFAs are more stable than PUFAs, making them preferable for long-term storage. PUFAs, while essential for plant stress responses and maintaining membrane fluidity, are more prone to oxidation due to their higher reactivity. Therefore, in terms of extending shelf life, a higher percentage of SFAs and MUFAs would be more beneficial compared to PUFAs.

### 3.5. Tocopherol Content

α- and γ-tocopherols are both forms of vitamin E, with α-tocopherol being the most abundant in hazelnut oil (Table 4), consistent with the findings reported in the literature [36].

Although there are visible differences in the levels of tocopherols between high, medium, and low plant density, these differences were not statistically significant, suggesting that other factors (such as genetics, environmental conditions, or harvest timing) might have a greater influence on their concentration than plant density.

### 3.6. Principal Component Analysis

**Figure 2:**
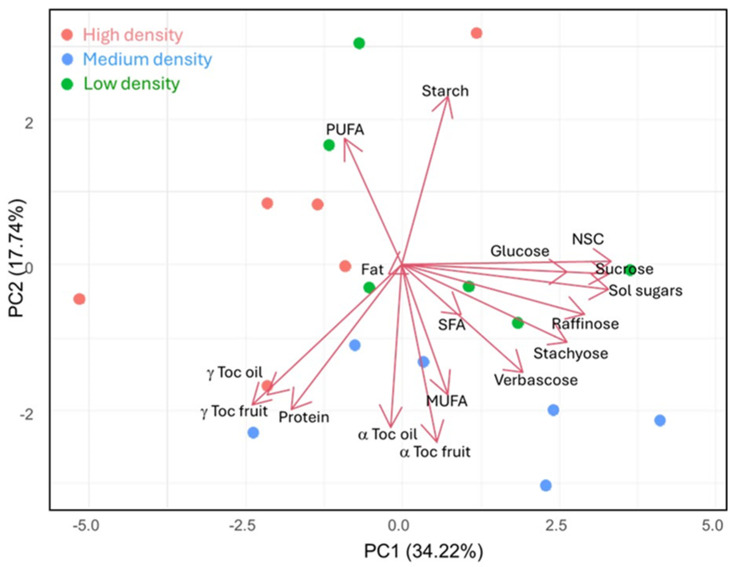
Scatter plot of the scores of samples from a high planting density (red colored), medium planting density (blue colored), and low planting density (green colored) on the two-dimensional plane defined by PC1 and PC2.

The PCA results revealed clear differences in hazelnut fruit quality traits, with distinct variations visible across the different planting densities. Notably, the samples from the medium-density planting were more clearly distinguished from those of the low- and high-density groups, highlighting the influence of planting density on fruit quality (Figure 2).

PC1, which accounts for 34.22% of the total variance, was primarily influenced by NSC, total soluble sugars, and sucrose content. These compounds are positioned on the right side of the plot, indicating that sugar metabolism played a significant role in discriminating samples. PC2, explaining 17.74% of the variance, showed a clear contrast between starch content on one side and α-tocopherol on the opposite side, suggesting opposing metabolic trends: starch accumulation was more prominent in certain samples from low-density planting system, whereas α-tocopherol synthesis was higher in samples from medium-density planting system. Medium-density samples exhibited higher levels of α-tocopherols and MUFAs, while their starch and PUFA content is lower. This aligns with findings from [37], who reported that increased light exposure enhances α-tocopherol and MUFA synthesis. In the context of medium-density hazelnuts, moderate light conditions likely optimized the production of α-tocopherol and MUFAs, protecting plants from oxidative stress and preserving oleic acid, the major constituent of MUFAs [38]. The alignment of the vectors for α-tocopherol and MUFAs on the PCA plot reinforces this association between light exposure, antioxidant production, and fatty acid composition [39].

In contrast, both low-density and high-density samples tended to have lower concentrations of α-tocopherol and MUFAs but a higher starch and PUFA content. This could be due to limited light availability at higher planting densities and nutrient competition or the light and therefore also thermal excess at low planting densities, prompting the plants to prioritize energy storage (as starch) and PUFA production, which may help to maintain cell membrane fluidity under suboptimal conditions.

The low-planting-density samples, positioned on the right side of the biplot, are notable for their elevated levels of NSC, soluble sugars, and sucrose, indicating enhanced carbohydrate metabolism likely due to better light penetration and reduced competition. Conversely, high-planting-density samples, located on the left side, showed increased levels of γ-tocopherols but lower sugar content, reflecting a possible adaptive response to lower light availability, where γ-tocopherols function as antioxidants to counteract oxidative stress caused by limited light and overcrowded conditions.

## 4. Conclusions

This study highlighted the significant impact of planting density on the light penetration, yield, and quality traits of grafted hazelnuts from the cultivar ‘Tonda Francescana^®^’ on *Corylus colurna* L.’ rootstocks during the sixth growing season. The results indicate that, also in young grafted plants, medium-density planting offers an optimal balance between light penetration, canopy development, and yield efficiency. Specifically, medium planting density promoted the synthesis of MUFAs in conjunction with key antioxidants such as α-tocopherol, which contributed to improved kernel quality and enhanced health benefits. This balance optimized the metabolic processes of the plant, resulting in higher antioxidant activity that protects the kernel from oxidative stress and helps to preserve oleic acid, a major MUFA. In contrast, high-density planting, while offering the highest yield per hectare due to increased tree numbers, although only 38.7% more, led to reduced light availability and to a shift towards energy storage in the form of starch and higher PUFA content. Low-density planting, though providing increased light penetration, resulted in a lower overall yield, suggesting that excessive light may not always be beneficial for optimal nut production and quality. These findings underscore the importance of carefully managing planting density in hazelnut orchards to maximize both yield and quality, with medium density emerging as the most favorable for maintaining a balanced metabolic response. This study also highlights the potential of chemometric analysis, such as PCA, in discerning complex metabolic trends linked to planting density, offering valuable insights for future breeding and crop management strategies.

## Figures and Tables

**Table 1:** Effects of plant density on canopy volume, light penetration, and yield in hazelnut from Tonda Francescana^®^ cultivar grafted on no-suckering rootstock. Data are reported as mean values. Mean values followed by different letters are significantly different as per α < 0.05.

Plant Density	Canopy Volume (m^3^)	Light Penetration (%)	Yield (kg/Tree)	Yield Efficiency (kg/m^3^)	Yield (kg/ha)
high	3.5 b	19.2 b	1.16 b	0.34 a	2898 a
medium	4.4 a	46.9 a	1.67 a	0.39 a	2089 b
low	3.7 ab	53.0 a	1.32 b	0.36 a	822 c

**Table 2:** Effects of density of planting on the protein, fat, and NSC contents in hazelnut fruit (% of dry matter). Data are reported as mean values ± standard deviation. Mean values followed by different letters are significantly different as per α < 0.05. Protein, fat and NSC contents are expressed as % of dry matter.

Tree Density	Protein	Fat	Glucose	Sucrose	Raffinose	Stachyose	Verbascose	Starch	Solubles	Tot NSC
high	18.9 ± 1.2	63.3 ± 2.2	0.009 b	3.5 ± 0.4	0.17 ± 0.01	0.65 ± 0.09	0.009 ± 0.007	0.25 b	4.4 ± 0.4	4.6 ± 0.5
medium	19.2 ± 0.8	64.8 ± 1.6	0.014 a	4.4 ± 0.8	0.21 ± 0.03	0.77 ± 0.13	0.007 ± 0.001	0.24 b	5.4 ± 0.9	5.7 ± 0.9
low	18.1 ± 0.9	64.5 ± 0.6	0.011 b	4.3 ± 0.7	0.20 ± 0.01	0.77 ± 0.12	0.006 ± 0.001	0.53 a	5.4 ± 0.8	5.9 ± 0.8

**Table 3:** Effect of density of planting on fatty acid composition. Data are reported as mean values. Mean values followed by different letters are significantly different as per α < 0.05.

Plant Density	SFAs (%)	MUFAs (%)	PUFAs (%)
high	9.5 b	80.2 b	10.3 a
medium	9.9 a	81.4 a	8.7 b
low	9.8 a	79.9 b	10.1 ab

**Table 4:** Effect of density of planting on γ- and α-tocopherol content analyzed as mg/100 g oil and as mg/100 g of fruit dry weight (dw). Data are reported as mean values ± standard deviations.

Plant Density	γ Tocopherol (mg/100 g Oil)	α Tocopherol (mg/100 g Oil)	α + γ Tocopherol (mg/100 g Oil)	γ Tocopherol (mg/100 g dw)	α Tocopherol (mg/100 g dw)	α + γ Tocopherol (mg/100 g dw)
high	0.99 ± 0.20	10.5 ± 1.4	11.5 ± 1.5	0.43 ± 0.20	4.6 ± 1.9	5.1 ± 1.9
medium	0.73 ± 0.11	13.1 ± 1.3	13.8 ± 1.3	0.32 ± 0.14	5.7 ± 1.9	6.0 ± 1.9
low	0.64 ± 0.87	12.5 ± 1.5	13.1 ± 1.5	0.28 ± 0.08	5.5 ± 1.8	5.8 ± 1.8

## Data Availability

The original contributions presented in the study are included in the article, further inquiries can be directed to the corresponding author.

## References

[1] Silvestri C., Bacchetta L., Bellincontro A., Cristofori V. (2021). Advances in Cultivar Choice, Hazelnut Orchard Management, and Nut Storage to Enhance Product Quality and Safety: An Overview. J. Sci. Food Agric..

[2] Orem A., Yucesan F.B., Orem C., Akcan B., Kural B.V., Alasalvar C., Shahidi F. (2013). Hazelnut-Enriched Diet Improves Cardiovascular Risk Biomarkers beyond a Lipid-Lowering Effect in Hypercholesterolemic Subjects. J. Clin. Lipidol..

[3] Amaral J.S., Casal S., Seabra R.M., Oliveira B.P.P. (2006). Effects of Roasting on Hazelnut Lipids. J. Agric. Food Chem..

[4] Bacchetta L., Aramini M., Zini A., Di Giammatteo V., Spera D., Drogoudi P., Rovira M., Silva A.P., Solar A., Botta R. (2013). Fatty Acids and Alpha-Tocopherol Composition in Hazelnut (*Corylus avellana* L.): A Chemometric Approach to Emphasize the Quality of European Germplasm. Euphytica.

[5] Pourfarzad A., Mehrpour G.R. (2017). Health Benefits of Hazelnut. EC Nutr..

[6] Snelgar W.P., Manson P.J., Martin P.J. (1992). Influence of time of shading on flowering and yield of kiwifruit vines. J. Hortic. Sci..

[7] Tombesi S., Palliotti A., Poni S., Farinelli D. (2015). Influence of Light and Shoot Development Stage on Leaf Photosynthesis and Carbohydrate Status during the Adventitious Root Formation in Cuttings of *Corylus avellana* L.. Front. Plant Sci..

[8] Toscano S., Trivellini A., Cocetta G., Bulgari R., Francini A., Romano D., Ferrante A. (2019). Effect of Preharvest Abiotic Stresses on the Accumulation of Bioactive Compounds in Horticultural Produce. Front. Plant Sci..

[9] Karaosmanoğlu H. (2022). Lipid Characteristics, Bioactive Properties, and Mineral Content in Hazelnut Grown under Different Cultivation Systems. J. Food Process. Preserv..

[10] Ellena M., González A., Sandoval P., Marchant F. (2018). Advantages of High Density Hazelnut Orchards in South Chile. Acta Hortic..

[11] Loreti F., Morini S., Muleo R., Masetti C., Vitagliano C. (1993). Effect of solar radiation on some growth paremeters of peach fruits. Acta Hortic..

[12] Beyhan N. (2007). Effects of planting density on yield and quality characteristics of hazelnut (cv. Palaz) in a hedgerow training system. Can. J. Plant Sci..

[13] Cristofori V., Bertazza G., Bignami C. (2015). Changes in kernel chemical composition during nut development of three Italian hazelnut cultivars. Fruits.

[14] Hampson C.R., Azarenko A.N., Potter J.R. (1996). Photosynthetic rate, flowering, and yield component alteration in hazelnut in response to different light environments. J. Am. Soc. Hortic. Sci..

[15] Bignami C., Bertazza G., Bizzarri S., Bruziches A., Cammilli C., Cristofori V. (2005). Effect of High Density and Dynamic Tree Spacing on Yield and Quality of the Hazelnut Cultivar ‘tonda Gentile Romana’. Acta Hortic..

[16] Tombesi A. (1978). Effect of Light Penetration on High Density Filbert Planting.

[17] Fideghelli C., De Salvador F.R. (2009). World Hazelnut Situation and Perspectives. Acta Hortic..

[18] Rovira M. (2021). Advances in Hazelnut (*Corylus avellana* L.) Rootstocks Worldwide. Horticulturae.

[19] Portarena S., Gavrichkova O., Brugnoli E., Battistelli A., Proietti S., Moscatello S., Famiani F., Tombesi S., Zadra C., Farinelli D. (2022). Carbon Allocation Strategies and Water Uptake in Young Grafted and Own-Rooted Hazelnut (*Corylus avellana* L.) Cultivars. Tree Physiol..

[20] Me G., Valentini N., Caviglione M., Lovisolo C. (2005). Effect of Shade on Flowering and Yield for Two Different Hazelnut Training Systems. Acta Hortic..

[21] Anthony B.M., Minas I.S. (2021). Optimizing peach tree canopy architecture for efficient light use, increased productivity and improved fruit quality. Agronomy.

[22] Pannico A., Cirillo C., Giaccone M., Scognamiglio P., Romano R., Caporaso N., Sacchi R., Basile B. (2017). Fruit Position within the Canopy Affects Kernel Lipid Composition of Hazelnuts. J. Sci. Food Agric..

[23] Vinci A., Traini C., Portarena S., Farinelli D. (2023). Assessment of the Midseason Crop Coefficient for the Evaluation of the Water Demand of Young, Grafted Hazelnut Trees in High-Density Orchards. Water.

[24] Angell A.R., Mata L., de Nys R., Paul N.A. (2016). The protein content of seaweeds: A universal nitrogen-to-protein conversion factor of five. J. Appl. Phycol..

[25] Proietti S., Moscatello S., Riccio F., Downey P., Battistelli A. (2021). Continuous Lighting Promotes Plant Growth, Light Conversion Efficiency, and Nutritional Quality of *Eruca vesicaria* (L.) Cav. in Controlled Environment With Minor Effects Due to Light Quality. Front. Plant Sci..

[26] Mitsikaris P.D., Kokokiris L., Pritsa A., Papadopoulos A.N., Kalogiouri N.P. (2022). Investigating the Tocopherol Contents of Walnut Seed Oils Produced in Different European Countries Analyzed by HPLC-UV: A Comparative Study on the Basis of Geographical Origin. Foods.

[27] Peacock A.G., Mullen M.D., Ringelberg D.B., Tyler D.D., Hedrick D.B., Gale P.M., White D.C. (2001). Soil microbial community responses to dairy manure or ammonium nitrate applications. Soil Biol. Biochem..

[28] Jolliffe I.T., Cadima J. (2016). Principal component analysis: A review and recent developments. Philosophical transactions of the royal society A: Mathematical. Phys. Eng. Sci..

[29] Jahirul M.I., Rasul M.G., Brown R.J., Senadeera W., Hosen M.A., Haque R., Saha S.C., Mahlia T.M.I. (2021). Investigation of correlation between chemical composition and properties of biodiesel using principal component analysis (PCA) and artificial neural network (ANN). Renew. Energy.

[30] Portarena S., Gavrichkova O., Brugnoli E., Battistelli A., Proietti S., Moscatello S., Famiani F., Zadra C., Tombesi S., Farinelli D. (2023). Grafted Hazelnut: A Sustainable Agricultural Practice to Face Summer Stressful Conditions. Acta Hortic..

[31] Ryugo K., Marangoni B., Ramos D.E. (1980). Light intensity and fruiting effects on carbohydrate contents, spur development, and return bloom of ‘Hartley’ walnut. J. Am. Soc. Hortic. Sci..

[32] Pretzsch H. (2014). Canopy space filling and tree crown morphology in mixed-species stands compared with monocultures. For. Ecol. Manag..

[33] Kumar R., Sharma S., Pathania V. (2013). Effect of shading and plant density on growth, yield and oil composition of clary sage (*Salvia sclarea* L.) in north western Himalaya. J. Essent. Oil Res..

[34] Gülsoy E., Kaya E.D., Türkhan A., Bulut M., Koyuncu M., Güler E., Sayın F., Muradoğlu F. (2023). The Effect of Altitude on Phenolic, Antioxidant and Fatty Acid Compositions of Some Turkish Hazelnut (*Coryllus avellana* L.) Cultivars. Molecules.

[35] Upchurch R.G. (2008). Fatty Acid Unsaturation, Mobilization, and Regulation in the Response of Plants to Stress. Biotechnol. Lett..

[36] Momchilova S.M., Taneva S.P., Zlatanov M.D., Antova G.A., Angelova-Romova M.J., Blagoeva E. (2017). Fatty Acids, Tocopherols and Oxidative Stability of Hazelnuts during Storage. Bulg. Chem. Commun..

[37] Munné-Bosch S., Alegre L. (2002). The Function of Tocopherols and Tocotrienols in Plants. Crit. Rev. Plant Sci..

[38] Ljubic A., Holdt S.L., Jakobsen J., Bysted A., Jacobsen C. (2021). Fatty acids, carotenoids, and tocopherols from microalgae: Targeting the accumulation by manipulating the light during growth. J. Appl. Phycol..

[39] Conde T.A., Neves B.F., Couto D., Melo T., Neves B., Costa M., Silva J., Domingues P., Domingues M.R. (2021). Microalgae as sustainable bio-factories of healthy lipids: Evaluating fatty acid content and antioxidant activity. Mar. Drugs.

